# Intradermal and Subcutaneous Botulinum Toxin Type A Injections Do Not Differ in the Induction of Neutralizing Antibody Formation

**DOI:** 10.3390/toxins18060245

**Published:** 2026-05-27

**Authors:** Stefanie Honndorf, Jessica Moser, Klaus Fink

**Affiliations:** Neurotoxin & Biotechnology Development, Merz Therapeutics GmbH, Eckenheimer Landstr. 100, 60318 Frankfurt, Germany

**Keywords:** botulinum toxin type A, BoNT/A, neutralizing antibodies, immunogenicity, intradermal, subcutaneous, mouse digit abduction score

## Abstract

The neurotoxin botulinum toxin type A (BoNT/A), produced by the bacteria *Clostridium botulinum*, is commonly injected intramuscularly (IM) for the management of chronic muscle hyperactivity, such as post-stroke spasticity. New indications such as peripheral neuropathic pain require alternative routes of BoNT/A administration, such as subcutaneous (SC) or intradermal (ID). While IM BoNT/A injections may elicit anti-drug-antibodies (ADAs), their occurrence following SC or ID administration is unknown. Therefore, we investigated whether repeated SC or ID injections of 150 kDa BoNT/A can elicit ADAs in a dose-dependent manner, and whether these differ depending upon the route of administration. Mice were injected 5 times ID or SC with 150 kDa BoNT/A or, for higher doses, inactive mutant BoNT/A (DRBoNT/A) or inactivated toxoid (IA-BoNT/A). Total ADAs were analyzed by an immunoassay and the subgroup of neutralizing ADAs by an in vivo digit abduction score (DAS) assay following a challenge of 0.6 U BoNT/A IM. DRBoNT/A and IA-BoNT/A injections elicit ADAs (22.7 U/mL vs. 460.5 U/mL for ID; 4.7 U/mL vs. 339.4 U/mL for SC), while therapeutic doses of 150 kDa BoNT/A do not. Whereas mice with repeated 150 kDa BoNT/A injections at therapeutic dose show an unrestricted DAS of 3.7 (ID) or 3.4 (SC), mice injected repeatedly with 1.8 µg/kg DRBoNT/A or 500 µg/kg IA-BoNT/A show only a minimal DAS of ≤0.7, indicating a high titer of neutralizing ADAs. No differences were observed between administration routes. Accordingly, repeated ID or SC injections of pure 150 kDa BoNT/A at therapeutic doses fail to induce ADA formation in mice. On the other hand, DRBoNT/A ID injections induce higher ADA concentrations than SC, but generate similar amounts of neutralizing ADAs. IA-BoNT/A injections induce ADAs and neutralizing ADAs similarly after ID and SC injections. ADA development at intermediate BoNT/A doses can be higher after ID injection, but does not lead to differences in neutralizing ADAs. Our data demonstrate that the antibody response to botulinum toxin depends predominantly on the protein load, and less on the administration route.

## 1. Introduction

Botulinum neurotoxin type A (BoNT/A) is used for the treatment of neurologic and aesthetic conditions with involuntary muscle hyperactivity, such as dystonia or spasticity. BoNT/A blocks acetylcholine release from nerve terminals at the neuromuscular junction, resulting in reversible chemical paralysis [1,2,3,4]. In clinical therapy, BoNT/A-induced muscle paralysis lasts for several months, but still requires repeated dosing to sustain the therapeutic effect [1,2,3,5,6,7]. This repeated exposure to the large bacterial protein BoNT/A can induce an immune response leading to the formation of anti-drug-antibodies (ADAs), including the sub-group of neutralizing antibodies (NAbs), which reduce the treatment efficacy [8,9,10,11]. The development of neutralizing antibodies after repeated treatment cycles of BoNT/A is well documented for a small group of patients [12,13]. This NAbs development was especially prominent after treatment with early BoNT/A products with higher toxin and complexing protein content (50 pg/U; “original” Botox pre-1998) than current, more purified products with lower protein content (4.4 pg/U; Xeomin) [12,13]. Irrespectively of toxin content, the specific role of the hemagglutinins or of inactivated BoNT/A in the elicitation of ADAs and NAbs still requires clarification.

Clostridia express the poly-cistronic botulinum neurotoxin genes to generate the protein complex, which consist of a 150 kDa neurotoxin and associated non-toxic accessory proteins (NAPs). The 150 kDa pro-toxin is catalytically cleaved to generate a di-chain consisting of a 100 kDa heavy chain and the catalytically active 50 kDa light chain connected by a disulfide bond [14]. The NAPs consist of hemagglutinin and non-toxic non-hemagglutinin proteins [15]. Those proteins spontaneously associate with the 150 kDa core neurotoxin [16] upon co-expression in the Clostridia. The non-toxic non-hemagglutinin (NTNH) shields the 150 kDa toxin from the low pH in the stomach, while the hemagglutinins (HA) enhance the gut mucosal uptake of the toxin [14,17,18]. However, NAPs may also stimulate the immune system [19]; in particular, HA33 has been proposed to be an immunoadjuvant [20]. Biological neutralization will occur in vivo if the ADAs interfere with the heavy chain binding site of BoNT/A for Gt1b or SV2c [21,22,23,24]. If their titers are high enough, these NAbs can inhibit the biological activity of the toxin completely [24,25]. Accordingly, and as with other immunogens, the risk for ADA and NAb formation towards BoNT/A is increased by high doses, short injection intervals, the duration of treatment, the formulation [26,27], and the amount of inactive toxin in the BoNT/A product. Inactive BoNT/A increases the total antigen load and thus, unnecessarily, the antigenic potential of neutralizing antibody generation, while lacking any or displaying only a diminished therapeutic effect [28]. This occurrence of immunogenicity [29] can lead to partial or complete secondary treatment failure [30].

The first commercially available Botulinum neurotoxin type A products contained the 150 kDa BoNT/A, NAPs, and excipients such as human serum albumin. However, now a growing number of BoNT/A products are becoming commercially available containing only the 150 kDa toxin and excipients, i.e., products that are free of the complexing proteins [12].

In clinical studies, 150 kDa BoNT/A products lacking complexing proteins displayed very low NAb formation in comparison to those additionally containing complexing proteins. The prevalence of NAbs in sera of cervical dystonia (CD) patients analyzed with the mouse hemidiaphragm assay (HDA) [31] was zero if they were treated with a BoNT/A product free of complexing proteins (IncobotulinumtoxinA; 0/70 patients). In comparison, patients treated with a BoNT/A product containing NAPs (Abo-or OnabotulinumtoxinA) displayed significant generation of NAbs (84/584 patients) [27]. The mean duration of BoNT/A treatment was 7.9 years. Other studies also show that BoNT/A products lacking complexing proteins elicit little or no ADA and NAbs formation [32,33]. A meta-analysis of 43 clinical studies from 2000 to 2020 on 8833 patients treated with BoNT/A for spasticity, cervical dystonia, blepharospasm, urological diseases, hyperhidrosis, or glabellar frown lines found an NAbs incidence of 0.3% following treatment with a BoNT/A product free of complexing proteins [34]. However, no information was provided in the publication on whether the patients displaying NAbs against BoNT/A had been pretreated with a BoNT/A product containing complexing proteins prior to their study enrollment. A further study was conducted on 613 children and adolescents suffering from spasticity or sialorrhea, which were treated with a BoNT/A preparation free of complexing proteins. In this study, no BoNT/A treatment-naïve patient generated NAbs. Only patients who were already NAb-positive prior to treatment or who had been pretreated with OnabotulinumtoxinA and/or AbobotulinumtoxinA before they were enrolled in the study displayed or developed NAbs (*n* = 7 for spasticity/*n* = 3 for sialorrhea). No secondary treatment failure was observed [35].

Currently, IncobotulinumtoxinA is approved for intramuscular and intraglandular use. Alternative routes like subcutaneous (SC) or intradermal (ID) injection may expand the therapeutic potential for clinical use, particularly in the management of neuropathic pain [36] or in dermatological applications [37]. Understanding the immunogenic potential of the administration routes is therefore critical for optimizing long-term treatment outcomes.

Therefore, in this study, we examined the immunogenicity of 150 kDa BoNT/A without complexing proteins following repetitive ID or SC injections in mice. To investigate the protein load/dose–response relationship, active 150 kDa BoNT/A was used for low doses, and an inactive, recombinant BoNT/A mutant (DRBoNT/A) was utilized for higher dose injections. An inactivated IA-BoNT/A toxoid combined with the adjuvant Alhydrogel was additionally applied as the highest dose and served as positive control for the immune reaction in mice. Our goal was to investigate whether repeated ID or SC administration of BoNT/A leads to the formation of ADAs and Nabs, as well as whether they affect the biological effect of BoNT/A-paralysis. The degree of resulting paralysis was measured with the mouse digit abduction score (DAS) following a single IM challenge injection with 0.6 units of active BoNT/A on day 105 of the study, 3 days prior to termination.

## 2. Results

### 2.1. Study Design

**Figure 1:**
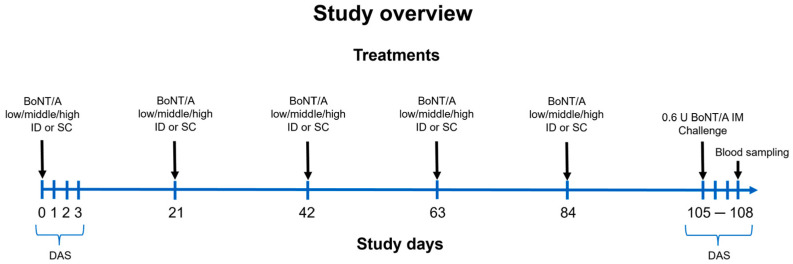
Graphical overview of the study design showing the immunization treatments over time. The digit abduction score (DAS) was determined on day 0 before treatment and on days 1–3 and 105–108 after treatment start. The DAS scorings on days 0 and 1–3 showed the baseline of the animals. The DAS scorings on days 106–108 showed the local paralysis in response to a BoNT/A IM injection on day 105 to investigate whether the BoNT/A ID or SC treatments caused neutralizing anti-drug-antibodies. On the last study day (day 108), blood serum was prepared for the fluorescence immunoassay. Botulinum toxin type A, BoNT/A; DRBoNT/A, detoxified recombinant, double mutant BoNT/A; IA-BoNT/A, formaldehyde inactivated botulinum toxin subtype A; ID, intradermal; SC, subcutaneous; t, treatment; IM, intramuscular.

This study was designed around five SC or ID injections of BoNT/A at three increasing doses. ADAs were determined in serum at the end on day 108. NAbs were determined in vivo by a DAS on days 106–108 after BoNT/A IM injection (challenge) on day 105. The initial DAS on days 0–3 was conducted to evaluate (1) the baseline digit abduction reflex of the animals and (2) potential paralytic effect of the first ID or SC treatment (Figure 1).

### 2.2. ADA Formation After Repetitive ID or SC Injection of BoNT/A

The baseline measurement on day 0 revealed no ADAs against BoNT/A in sera of mice in any group. BoNT/A (150 kDa, without complexing proteins)-injected mice (ID or SC) remained ADA-negative throughout the study.

**Figure 2:**
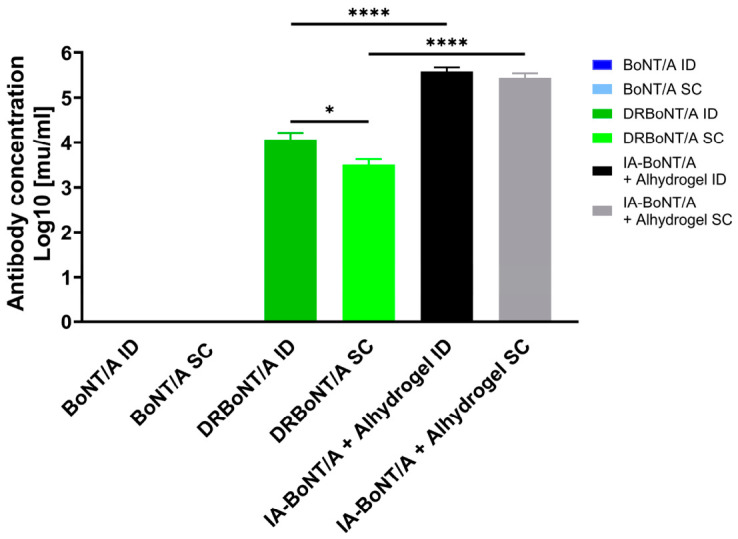
Impact of repeated SC or ID dosing with BoNT/A, DRBoNT/A, or IA-BoNT/A toxoid plus Alhydrogel on the development of BoNT/A ADAs. Mean concentration of ADAs per group at day 108. BoNT/A-treated groups did not show ADA development. DRBoNT/A ID-treated mice developed significantly lower ADA concentrations compared to SC treatment. The concentration of ADAs was highest after IA-BoNT/A injection, reflecting a dose dependency. There was no difference among toxoid groups. Ordinary two-way ANOVA followed by Tukey’s multiple comparisons test (* *p* < 0.05 DRBoNT/A ID vs. DRBoNT/A SC; **** *p* < 0.0001 DRBoNT/A ID or SC vs. IA-BoNT/A ID or SC. Data after log 10 transformation of mU/mL concentration. Mean + SEM, *n* = 10).

After injection of DRBoNT/A ID or SC at higher dose, we found ADAs in the serum with higher Ab titers after ID injection (22.7 ± 11.1 U/mL for ID vs. 4.7 ± 1.4 U/mL for SC, *p* < 0.05, respectively). The mean ADA concentration in the DRBoNT/A groups was, however, significantly lower than in the highest dose IA-BoNT/A groups, demonstrating a dose-dependent ADA generation (22.7 ± 11.1 vs. 460.5 ± 86.1 U/mL for ID and 4.7 ± 1.4 vs. 339.4 ± 60.4 U/mL for SC; *p* < 0.05, DRBoNT/A vs. IA-BoNT/A). Comparing both injection routes per test substance, the ADA concentration was higher after ID injection of DRBoNT/A compared to SC injection (22.7 ± 11.1 for ID vs. 4.7 ± 1.4 U/mL for SC; *p* < 0.05). No difference in ADA concentration was observed between ID and SC injection of IA-BoNT/A (*p* > 0.05; Table 1 and Figure 2):

### 2.3. Impact of Repetitive BoNT/A Injections on Digit Abduction Score

BoNT/A was injected into the gastrocnemius muscle of mice to assess muscle paralysis using the DAS assay. This assay is a common experimental procedure to investigate the duration of action of BoNT/A products or to compare their potency in vivo [38,39]. In this study, the DAS was performed to investigate if repetitive ID or SC injections of BoNT/A, DRBoNT/A, or IA-BoNT/A plus Alhydrogel over 3 months caused a lower paralytic effect of BoNT/A after a single “challenge” IM injection on day 105, which would be indicative of NAbs.

**Figure 3:**
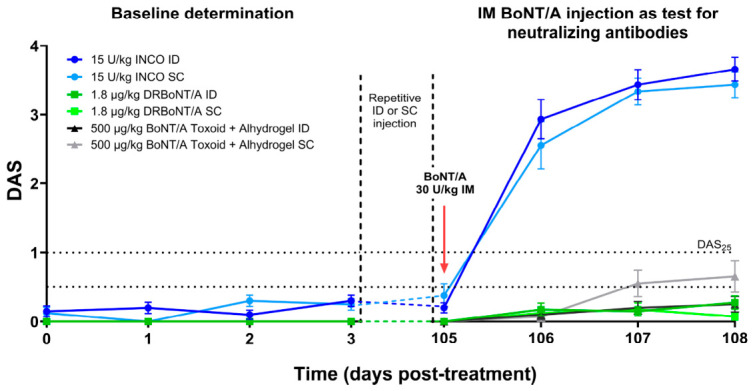
Effect of repeated SC or ID injection of BoNT/A, DRBoNT/A, or IA-BoNT/A combined with Alhydrogel on digit abduction score (DAS). Paralysis in mice after administration of 30 U/kg BoNT/A IM on day 105 detected via the mouse digit abduction score (DAS). A DAS response curve was demonstrated in BoNT/A groups from days 0 to 3 and 105 to 108 compared to treatment with DRBoNT/A or IA-BoNT/A. No DAS was performed during the repetitive treatment phase (black vertical dashed lines) between day 3 and 105. The paralytic effect did not differ among BoNT/A administration routes (two-way RM ANOVA followed by Šídák’s multiple comparisons test ID vs. SC *p* > 0.05). DRBoNT/A or IA-BoNT/A groups did not show a paralytic effect or any differences between administration routes (two-way RM ANOVA followed by Šídák’s multiple comparisons test DRBoNT/A or IA-BoNT/A ID vs. SC *p* > 0.05, respectively). Mean ± SEM, *n* = 10. Horizontal dotted black lines show the DAS25 and DAS12.5 levels for orientation.

From day 0 until day 3, all mice displayed full digit abduction (DAS ≤ 0.3). After the IM injection of 30 U/kg BoNT/A on day 105, only the BoNT/A-treated groups displayed a mean peak DAS of 3.7 and 3.4, showing almost full paralysis in the ID and SC group, respectively (days 106–108, Table 2 and Figure 3).

In the DRBoNT/A and IA-BoNT/A groups, however, the mean DAS in response to the BoNT/A test injection on day 105 was ≤0.7, indicating essentially no paralysis. Independent of BoNT/A, DRBoNT/A, or IA-BoNT/A plus Alhydrogel injection series, the DAS response was not different between ID and SC.

### 2.4. Impact of Repetitive BoNT/A Injections on Body Weight

**Figure 4:**
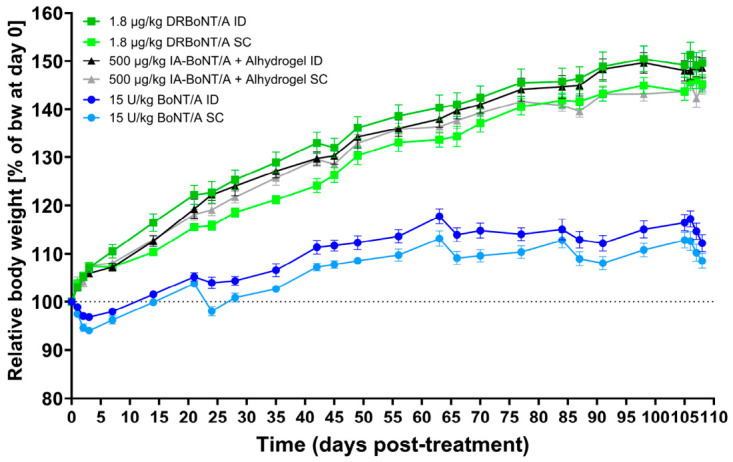
Effect of repeated SC or ID injection of BoNT/A, DRBoNT/A, or IA-BoNT/A combined with Alhydrogel on body weight. BoNT/A-treated groups showed an initial body weight decline compared to day 0, which was higher in the SC group, followed by a comparable increase in weight after day 3 (two-way RM ANOVA followed by Šídák’s multiple comparisons test ID vs. SC *p* < 0.05). There was no loss of body weight after DRBoNT/A and IA-BoNT/A combined with Alhydrogel injection via ID and SC routes (two-way RM ANOVA followed by Šídák’s multiple comparisons test DRBoNT/A or IA-BoNT/A ID vs. SC *p* > 0.05, respectively). Mean ± SEM, *n* = 10.

After the first injection of BoNT/A on day 0, a mean body weight decrease of 3.2% in the ID injected group (day 3) and 6.0% in the SC injected group (day 3) was observed (ID vs. SC *p* < 0.05). Taking this body weight loss into account, the BoNT/A-treated mice showed a comparable increase in weight in both groups after day 3 (maximum weight gain 17.8% in ID group vs. 13.2% in SC group, Figure 4). After the subsequent BoNT/A injections, transient decreases in body weight were again observed.

Mice treated with DRBoNT/A or IA-BoNT/A, irrespective of ID or SC injection, gained body weight at similar rates between 45 and 51% without any periods of weight loss.

Administration of 0.6 U BoNT/A on day 105 IM led again to body weight decrease of 4.3% in the BoNT/A ID and BoNT/A SC groups (day 107 vs. day 105). No weight loss was observed in the higher-dosed groups. Interestingly, the body weight increase of the BoNT/A-treated animals slowed down from injection to injection, and the animals did not gain further body weight at all after the fourth and following injections, whereas the animals treated with the high-dosed inactive DRBoNT/A or the inactivated IA-BoNT/A toxoid continuously gained body weight, and did not even lose body weight after the single IM injection of BoNT/A on day 105.

## 3. Discussion

Repetitive BoNT/A injections for the treatment of neurologic or aesthetic conditions such as spasticity or dystonia [1,2,3,4,6] bear the risk of inducing ADAs and, as part of this, NAbs, which may result in reduced treatment efficacy [8,9,10,11,40,41,42]. Among other factors, the risk depends on the administration route [26,27,43]. While the potential formation of NAbs after repetitive IM injection of BoNT/A free of complexing proteins was studied in several clinical trials [44], the SC and ID routes may entail different risks for ADAs or NAbs generation, and have not been studied so far. They are, however, becoming more important because BoNT/A SC is currently in clinical development for peripheral neuropathic pain [36] and scar neuroma pain [45]. After SC injection, BoNT/A is rapidly drained into the blood circulation and becomes heavily diluted. After ID injection, BoNT/A remains confined for a short time in the injected intradermal bleb before it is at least partially taken up by professional antigen-presenting cells, such as dermal Langerhans cells, which process and present it as an antigen and activate the immune response [46]. Therefore, understanding the immunogenic potential after repeated ID or SC BoNT/A administrations is crucial for estimating the risk of long-term ID or SC treatment as considered for peripheral neuropathic pain treatment or for migraine prevention [47].

Here, BoNT/A immunogenicity after repeated ID or SC administration was compared in vivo in mice. Groups 1 and 2 were repetitively (5 times, 21 days interval) injected with therapeutic doses of 150 kDa BoNT/A, i.e., the lowest protein load. Groups 3 and 4 were repetitively injected with a higher dose of 150 kDa BoNT/A. To allow for the investigation of these otherwise lethal BoNT/A protein doses, a full-length inactive recombinant BoNT/A mutant (DRBoNT/A) was used, with a double amino acid exchange in the active center of the light chain, which still comes with the structural characteristics close to native toxin, but with 5 orders of magnitude lower catalytic activity [48,49]. Groups 5 and 6 were injected with an even higher BoNT/A dose, considered as a positive control for ADA generation. To accomplish this, inactivated BoNT/A toxoid (IA-BoNT/A, denatured) together with an immune adjuvant (Alhydrogel) was injected. Thus, three increasing BoNT/A doses could be administered ID or SC. The totality of ADAs was finally determined by ELISA. To assess the formation of NAbs, a DAS was performed at the end of the immunization period, as a functional assay. On day 105 of the study, 30 U/kg 150 kDa BoNT/A was injected into the gastrocnemius muscle of mice and the DAS was scored on the following days 106–108. This dose caused a mean peak DAS score of 3.4 in previous murine studies [50].

No ADAs were detected in the sera of mice treated with BoNT/A via the ID or SC route, which is consistent with the unrestricted paralytic effect in the DAS.

After ID or SC injection of the inactive mutant DRBoNT/A, we found 22.7 U/mL and 4.7 U/mL of ADAs in the sera, respectively. The lack of paralysis in the DAS indicates a significant concentration of NAbs in these animals. The ADA concentration was 2.6 times higher after ID injection compared to SC application (*p* < 0.05). The significantly higher proportion of ADAs after ID administration can be attributed to the more efficient pathway for antigen uptake and presentation in the dermis than in the subdermal compartment. However, the higher ADA concentration after ID injection did not lead to a reduced DAS score, indicating similar NAb titers after both injection routes. In a former study in mice, we injected DRBoNT/A (1.8 µg/kg) at the same dose IM four times every three weeks. ADAs were detected in 3 out of 10 mice at study end. In addition, we detected 25% decrease in paralysis in those mice demonstrating the presence of NAbs. This shows that even after IM injection into the muscle compartment, NAbs may occur after injection of a high BoNT/A protein load. However, the frequency is 70% lower compared to ID or SC injection.

In the sera of the IA-BoNT/A-injected mice, the ADA concentrations were significantly higher compared to the DRBoNT/A groups with 460.5 U/mL vs. 22.7 U/mL for ID and 339.4 vs. 4.7 U/mL for SC (*p* < 0.05 DRBoNT/A vs. IA-BoNT/A). Considering the dose of BoNT/A, which was 278 times higher in the IA-BoNT/A groups compared to DRBoNT/A groups (500 µg/kg vs. 1.8 µg/kg), the formation of ADAs appears to be dose-dependent. However, in addition to the higher dose, the toxoid injection contained Alhydrogel as an adjuvant, which makes it difficult to attribute the stronger immune reaction to the dose or to the adjuvant. The Ab concentrations did not differ between ID and SC toxoid groups.

In the two BoNT/A-treated groups, no ADAs were found, and in the functional assay, the mean peak DAS responses were 3.7 and 3.4 in the ID and SC group, respectively, indicating the absence of NAbs. The maximum paralysis score would have been 4.0. In this paradigm, a nearly maximum paralytic response was observed after 30 U/kg 150 kDa BoNT/A injected IM. DRBoNT/A and IA-BoNT/A injection led to a mean DAS of ≤0.7, indicating an almost complete inhibition of the paralytic BoNT/A effect by NAbs. Moreover, the majority of mice in those groups showed a DAS of 0 (DRBoNT/A 17/20 vs. IA-BoNT/A toxoid 12/20, Table 1). This finding can be attributed to the presence of NAbs in the right gastrocnemius muscle, which bound the IM-injected BoNT/A, preventing its action on the neuromuscular endplate. Neither with DRBoNT/A nor with IA-BoNT/A plus adjuvant was a difference in DAS response observed when comparing ID and SC groups, indicating that the level of NAbs was not affected by these injection routes. The ID and SC injection of DRBoNT/A and IA-BoNT/A led to a complete loss of the paralytic effect in the DAS, indicating that both injection routes and both compounds/doses led to the development of NAbs titers high enough to fully prevent the effect of the BoNT/A dose in the muscle.

We observed a transient decrease in body weight gain after BoNT/A administration (15 U/kg ID or SC or 30 U/kg IM) when compared to the four groups injected with inactive BoNT/A. This phenomenon is in line with our own and other observations after IM injection of BoNT/A between 20 and 40 U/kg [50,51,52,53]. It has been attributed to a decrease in locomotion and to immobility compared to placebo treatment [39]. The impaired mobility due to the local muscle relaxation was suggested to reduce food and water intake during the first days after BoNT/A injection [53].

We also found a transient decrease in body weight after the second, fourth, and fifth BoNT/A injection compared to the day before, which was more pronounced in the SC group than in the ID group (max. decrease for the SC group 5.7% on day 24 vs. max. decrease of 3.4% for the ID group on day 66). Repeated injection after 3 weeks leads to an accumulation of BoNT/A with more local muscle relaxation and decreased locomotion, leading to reduced food and water intake. It has to be inferred from the data that the animals injected with therapeutic doses of 150 kDa BoNT/A do not develop ADAs, and suffer from accumulating BoNT/A concentrations over the entire experimental period; the overlapping effect of BoNT/A is apparently so strong from the third injection that it prohibits further body weight gain. The body weight curve flattens out horizontally after the third injection, whereas the curve of the groups injected with high-dosed BoNT/A increased continuously, similar to the growth charts of untreated male BALB/c mice [54]. The higher decrease in the SC group may be due to the anatomical proximity of the subcutaneous tissue in which BoNT/A is injected to the subcutis, which is located directly above the muscle tissue, whereas the epidermis as the outer skin layer is more distant to the muscles [55]. If BoNT/A starts to spread from the injection site, the spread is supposed to be larger after SC injection, as the toxin is not constrained by the tight dermis tissue. The rapid drainage from the subcutaneous tissue into the circulation appears as serum peak, and may lead to more muscle relaxation and reduced food and water intake.

Taken together, the DAS results reflecting functionally relevant neutralizing ADAs correspond to the ELSIA results reflecting all ADAs. There was no difference in antibody formation or paralysis score after injection of BoNT/A or IA-BoNT/A after ID and SC injection. In addition, the significantly higher portion of ADAs after ID injection of the inactive DRBoNT/A did not lead to a functional difference in paralysis. Therefore, the risk for neutralizing ADA formation does not differ between repetitive ID and SC injection, at least at the BoNT/A concentrations applied in this study. Comparing our data from earlier investigations in which DRBoNT/A was injected IM, the ADA and NAb formation frequency was lower compared to ID and SC injection in this study. In a vaccine study, 40% dose of a Polio vaccine ID achieved the same ADA titer as the full dose IM [56], which has been observed with several other vaccines as well [57]. Whereas we demonstrated here similar or only slightly higher immunogenicity of ID vs. SC injection, vaccine studies found a substantially higher immunogenicity of ID vs. SC injection [57].

The immunogenicity of ID 150 kDa BoNT/A free of complexing proteins was previously investigated in rabbits [58] and compared to OnabotulinumtoxinA containing the NAPs. The authors concluded that BoNT/A free of complexing proteins shows low immunogenicity, even when using doses four-to-five times higher than the clinically recommended dose of 8 U/kg in patients with upper limb spasticity [59]. This finding was later confirmed by clinical trials with IncobotulinumtoxinA [44], and is also in line with our data in mice. We used in this study a BoNT/A dose of 15 U/kg because we found in a pilot study that 20 U/kg BoNT/A was not well tolerated by male BALB/cJBomTac mice when injected ID or SC every 3 weeks.

In addition to 150 kDa BoNT/A, a higher dose of the inactive mutant DRBoNT/A with 1.8 µg/kg, corresponding to 3.6 × 10^5^ U/kg or 7200 U per mouse (calculated with 5 pg/U), as well as an even higher dose of the inactivated IA-BoNT/A, with 500 µg/kg corresponding to 1 × 10^8^ U/kg or 2 × 10^6^ U per mouse, were injected. These doses are, respectively, 18 and 5000 times higher compared to the mean dose used in upper limb spasticity patients with 400 U [7], and are thus clinically not relevant. However, using these doses, we were able to demonstrate a dose-dependent ADA and NAb formation.

We used a fluorescence immunoassay to detect the anti-BoNT/A ADA concentrations. The determination of ADAs is dependent on the sensitivity and specificity of the assay. Therefore, the comparability of ADA concentrations in experimental or clinical studies using other analytical assays is limited.

The amount of detectable antibodies to BoNT/A should be critically viewed because the ADA concentration can be too low or not target the appropriate epitopes to become biologically inactivating and cause treatment failure. Therefore, we included the functional DAS assay to investigate if the level of NAbs would be sufficient to neutralize the paralytic effect. Antibody-mediated neutralization of BoNT/A can also be detected by the mouse protection assay (MPA) or the mouse hemidiaphragm assay (HDA) [60]. The HDA has a detection limit of ~0.0003 U/mL [11,61] and is 6–25 times more sensitive than the MPA [31,62], and provides more exact insights into the immunologic resistance or treatment failure.

Clinically relevant antibody levels represent a significant challenge for BoNT/A treatment. The threshold at which antibody concentrations significantly impact treatment efficacy remains elusive [63], but recombinant neurotoxins that have been developed, e.g., senrebotase [64], or are still in clinical development, e.g., IPN-10200 [65,66], have such low specific activities that the required therapeutic doses reach the protein load of the intermediate dose of DRBoNT/A in this study. Similarly, the protein doses injected when using BoNT/B can be 50 ng, which is even higher than the intermediate dose in this study [60].

Our results indicate that the immunogenic potential of repeated ID or SC injection of 150 kDA BoNT/A at therapeutic doses is low and does not induce ADA formation in our paradigm, which is consistent with the favorable safety and efficacy profile of BoNT/A free of complexing proteins after IM injection. However, increasing the dose, admittedly, far beyond clinically relevant doses causes anti-BoNT/A ADA formation, indicating a dose-dependent immune response to BoNT/A. At the medium dose (DRBoNT/A), more ADAs were developed after ID than after SC injection, which was no longer observed at the highest dose, presumably because the plateau of the humoral response was reached. Interestingly, there was no difference in NAbs between ID and SC. The dose threshold for ADA formation, which of course depends on more variables, lies between the low (1.3 pg) and the medium (36 ng) dose applied here, but judging from the clinical occurrence of NAbs at the low dose, it is closer to or beginning at the low dose. Since the threshold for the immune response is not known, patients should be treated with the minimum effective dose and protein amount; the recommended treatment intervals should not be shortened to minimize the risk for NAbs formation.

## 4. Conclusions

In this study, a BoNT/A immunization protocol was established to compare the immunogenic potential of ID and SD injection concentration-dependently. Injections of BoNT/A free of complexing proteins according to the immunization protocol did not induce neutralizing or non-neutralizing BoNT/A ADAs, irrespective of the administration route. However, injection of 18- or 5000-times higher doses than used therapeutically (8 U/kg) using the inactive mutant DRBoNT/AD or the toxoid IA-BoNT/A induced non-neutralizing and neutralizing BoNT/A ADAs in all animals, again irrespective of the administration route. The data are consistent with a dose-dependent response of the mammalian immune system, which underlines the importance of treating patients with the least possible BoNT/A protein amount. Depending on the dose, ID injection can induce more ADA development than SC, but ID or SC injection routes did, at the doses studied, surprisingly not differ in the functional test for NAbs.

## 5. Materials and Methods

### 5.1. Animals

Sixty 7-weeks-old male BALB/c mice weighing 20 g were included in the in vivo experiment (Taconic Biosciences, Ejby, Denmark). Mice were maintained on a 12 h light and 12 h dark cycle under regulated temperature at 22 ± 2 °C and relative humidity between 30 and 70% with unrestricted access to food and water. Acclimatization of mice took place for at least one week before initiation of experimental procedures. All animal experiments were performed according to the legislation of the European Communities Council Directive 2010/63/EU, and approval for all procedures was given by the respective local authority for the use of animals or scientific purposes (reference 2024-15-0201-01660, date 1 May 2024, Regional Council of Denmark; reference 2347-13-2023-39-G, date 10 April 2024, Regional Council of Brandenburg).

### 5.2. Botulinum Toxin A Formulations

The 150 kDa BoNT/A product free of complexing proteins (IncobotulinumtoxinA; Xeomin^®^, Merz Pharmaceuticals GmbH, Frankfurt, Germany), recombinant inactive double mutant botulinum neurotoxin type A termed DRBoNT/A (Prime Bio Inc., Dartmouth, MA, USA), and inactivated IA-BoNT/A toxoid (miprolab GmbH, Göttingen, Germany) combined with Alhydrogel^®^ adjuvant 2% (InvivoGen, Toulouse, France) were used in this study. Lyophilized BoNT/A in 100 U/vials was reconstituted using preservative-free saline (0.9% B.Braun Melsungen AG, Melsungen, Germany) with 0.2% human serum albumin (HSA; CSL Behring, Marburg, Germany). DRBoNTA/AD (0.38 mg/mL) was diluted in preservative-free saline with 0.2% HSA to 36 ng/mouse (1.8 µg/kg). It is a recombinant detoxified serotype A1 mutant lacking the endopeptidase activity critical for its toxicity and is used here as non-toxic surrogate for the native toxin [48].

The inactivated botulinum toxin subtype A1 (IA-BoNT/A) is a 150 kDa botulinum toxin A1 isolated from Clostridium botulinum bacteria culture and inactivated using formaldehyde (miprolab GmbH, Göttingen, Germany). IA-BoNT/A was mixed with Alhydrogel^®^ adjuvant 2% (Invivogen Europe, Toulouse, France) in 1:1 (*v*/*v*) before injection to obtain 10 µg toxoid/mouse (500 µg/kg).

### 5.3. Experimental Protocol

Mice were distributed to the treatment groups (*n* = 10/group) receiving either intradermal or subcutaneous injection. Before the test compounds were injected ID or SC, animals were put under isoflurane anesthesia in an induction chamber, which was sustained via a nose cone (5% isoflurane in 100% medical oxygen, 1 L/min) throughout the entire duration of the experimental procedure. The mice were administered with a mean dose of 0.3 U or 15 U/kg BoNT/A, 36 ng or 1.8 µg/kg DRBoNT/A, or 10 µg IA-BoNT/A combined with Alhydrogel (500 µg/kg). The injection site was shorn with an electric clipper (Aesculap AG, Tuttlingen, Germany). For the injections, a 100 µL Hamilton syringe (Hamilton Company, Reno, NV, USA) and a 30-gauge needle (B.Braun Melsungen AG, Melsungen, Germany) was used. The total volume for all test substances was 20 μL per animal. The procedure was repeated on study days 21, 42, 63, and 84. ID injection in mouse skin requires an experienced injector. A successful ID injection can easily be checked by the pale bleb under the skin surface at the injection site, whereas an SC injection remains mostly flat or appears as a subtle swelling. Body weight was detected before dosing and at predefined intervals until study end (day 108).

On study day 105, all mice were injected with 0.6 U BoNT/A into the right gastrocnemius muscle for functional assessment of NAbs by a DAS on days 106, 107, and 108. On day 108, mice were anesthetized with 5 vol.% isoflurane and whole blood of unconscious mice was removed by retro-orbital bleeding. Blood collection was performed using non-heparinized capillary (Minicaps 10 μL, Cat #6122439; VWR International GmbH, Darmstadt, Germany). Blood was collected into Z-clot activator 1.1 mL microtubes (Sarstedt Cat#41.1500.005, Nümbrecht, Germany). A volume of 400 μL serum was obtained per animal. Serum samples were incubated at room temperature (RT) for 30 min and then centrifuged at 10,000 g for 5 min at RT. Serum was stored at −80 °C until analysis.

### 5.4. Immunoassay to Determine Anti-BoNT/A Antibodies

An in-house-developed fluorescence immunoassay in ELISA format was used to quantify the antibody (ADA) concentration against the antigen BoNT/A in mouse serum samples. An immobilized antigen (Ag) and an Ag with a Biotin Tag were used to quantify the ADA concentrations of the various samples. A competitive assay was performed to verify the specific binding of these ADAs to BoNT/A in samples with a detectable ADA concentration.

To determine the ADA concentration, a 96-well plate (Maxi Sorp, Thermo Fisher #43110, Dreieich, Germany) was coated over night at room temperature (RT) with BoNT/A (1.25 µg/mL in phosphate buffered saline). The plate was washed (DELFIA Wash buffer, Revvity # 1244-114, Waltham MA, USA) and blocked with 1× RotiBlock (Carl Roth GmbH #A151.1, Karlsruhe, Germany) at RT for 1 h. A volume of 50 µL of each BoNT/A antibody standard concentration (Chiron Behring, PZN-2399673, Marburg, Germany) and sample was added into each well in quintuplicates for 30 min at RT after a second washing step. BoNT/A-Biotin (EZ-Link™ Sulfo NHS-LC-LC-Biotin, Thermo Fisher, A35358, Dreieich, Germany) was added and incubated for 2.5 h at RT. After a washing step, 100 µL/well Streptavidin-Eu (Revvity #1244-360, Waltham, MA, USA, 100 ng/mL in DELFIA Assay Buffer) was incubated for 1 h at RT. Before the 100 µL/well DELFIA Enhancement solution (Revvity #1244-105, Waltham, MA, USA) was incubated for 10 min at RT protected from light, another washing step was performed. Finally, the plate was analyzed with an excitation of 330/80 nm and an emission of 620/10 nm (Synergy H1, Agilent, Santa Clara, CA, USA).

The resulting relative fluorescence unit (RFU) values were analyzed using Excel (Microsoft Offics 365) and GraphPad Prism (version 9.5.1). A quadratic approximation of the standard curve RFU values was used to calculate antibody concentrations (mU/mL) of the samples. If a coefficient of variance (CV) of a standard concentration was higher than 20%, a Dixon outlier test was performed. If the Dixon test was positive, this well was excluded from the following calculations. If the CV or the recovery rate 75–125% criterion was still not fulfilled, up to two concentrations out of seven concentrations of the standard curve could be masked. The lower limit of detection was calculated as 3-times the standard deviation of the blank.

### 5.5. DAS Assay

A modified digit abduction score assay was performed, as previously described, to assess muscular function [50]. The DAS was determined for baseline on day 0 before the first treatment with ID or SC BoNT/A on that day. It was also determined on the following study days 1–3 in order to exclude the presence of local muscle paralysis in mice after BoNT/A ID or SC injection. After the five treatments with ID or SC BoNT/A, the DAS was performed on days 105–108. After the scoring on day 105, mice were injected with 0.6 U BoNT/A IM (=challenge injection) to induce muscle paralysis to be assessed by the DAS on days 106, 107, or 108. Each DAS was performed by the same experimenter.

### 5.6. Statistics

Body weight per group was expressed in percentage of weight change at each time point compared to the initial body weight (day 0). Body weight development over time was analyzed by two-way repeated-measures analysis of variance (RM ANOVA), followed by Šídák’s multiple comparisons post-hoc analysis. RM ANOVA was used to compare the mean DAS per dose and time point for assessment of treatment differences on the functional outcome over time. Comparisons between groups were analyzed using Šídák’s multiple comparisons test. For peak DAS comparisons, Kruskal–Wallis ANOVA followed by Dunn’s test was applied.

A two-way ANOVA followed by Tukey’s multiple comparisons test was used to compare NAb concentrations after ID vs. SC injection of test items.

All data are presented as individual data or group mean ± standard error of the mean (SEM). Statistical analyses were carried out using GraphPad Prism (version 9.5.1, GraphPad Software, San Diego, CA, USA). The threshold for the level of significance was set at α = 0.05.

## Figures and Tables

**Table 1:** Concentration of anti-drug-antibodies (ADAs) after ID or SC injections of BoNT/A, DRBoNT/A, and IA-BoNT/A on day 108.

Treatment	Immunization Dose/Mouse	No. of Animals	ADAs [mU/mL] Mean ± SEM	ADAs [Log10] of [mU/mL] Mean ± SEM
BoNT/A ID	0.3 U, corresponding to 1.3 pg	10	-	-
DRBoNT/A ID	36 ng	10	22,686 ± 11.1	4.1 # ± 0.2
IA-BoNT/A ID	10 µg	10	460,501 ± 86.1	5.6 * ± 0.1
BoNT/A SC	0.3 U, corresponding to 1.3 pg	10	-	-
DRBoNT/A SC	36 ng	10	4663 ± 1.4	3.5 ± 0.1
IA-BoNT/A SC	10 µg	10	339,350 ± 60.4	5.5 * ± 0.1

BoNT/A-treated groups did not show ADA development. DRBoNT/A ID-treated mice developed a significantly higher ADAs concentration compared to SC treatment (two-way ANOVA followed by Tukey’s multiple comparisons test # *p* < 0.05 DRBoNT/A ID vs. DRBoNT/A SC). The concentration of ADAs was highest after IA-BoNT/A injection, reflecting a dose dependency (* *p* < 0.0001 DRBoNT/A ID or SC vs. IA-BoNT/A ID or SC. Mean ± SEM, *n* = 10). There was no difference between ID and SC among the IA-BoNT/A groups. ADAs, anti-drug-antibodies.

**Table 2:** Digit abduction score (DAS) after IM injection of 0.6 U BoNT/A free of complexing proteins.

Treatment	Immunization Dose/Mouse	No. of Animals with DAS > 0.5 Out of 10 on Study Days	Mean Peak DAS	Study Day
BoNT/A ID	0.3 U, corresponding to 1.3 pg	10/10 on day 106	3.7 * ± 0.2	108
		10/10 on day 107		
		10/10 on day 108		
DRBoNT/A ID	36 ng	1/10 on day 106	0.3 ± 0.1	108
		0/10 on day 107		
		1/10 on day 108		
IA-BoNT/A ID	10 µg	0/10 on day 106	0.3 ± 0.1	108
		1/10 on day 107		
		1/10 on day 108		
BoNT/A SC	0.3 U, corresponding to 1.3 pg	10/10 on day 106	3.4 * ± 0.2	108
		10/10 on day 107		
		10/10 on day 108		
DRBoNT/A SC	36 ng	0/10 on day 106	0.2 ± 0.1	107
		1/10 on day 107		
		0/10 on day 108		
IA-BoNT/A SC	10 µg	1/10 on day 106	0.7 ± 0.2	108
		2/10 on day 107		
		3/10 on day 108		

Data are expressed as absolute numbers or mean ± SEM (Kruskal–Wallis ANOVA followed by Dunn’s test * *p* < 0.0001 BoNT/A ID vs. DRBoNT/A ID and IA-BoNT/A ID groups; * *p* < 0.0001 BoNT/A SC vs. DRBoNT/A SC and IA-BoNT/A SC groups).

## Data Availability

The original contributions presented in this study are included in the article. Further inquiries can be directed to the corresponding author.

## Abbreviations

The following abbreviations are used in this manuscript:

ADAs	Anti-drug-antibodies
BoNT/A	Botulinum toxin type A
CD	Cervical dystonia
CV	Coefficient of variance
DAS	Digit abduction score
DRBoNT/A	Detoxified recombinant, double mutant BoNT/A
ELISA	Enzyme-linked immunosorbent assay
HA	Hemagglutinin
HDA	Hemidiaphragm assay
HSA	Human serum albumin
IA-BoNT/A	Formaldehyde inactivated botulinum toxin subtype A1
ID	Intradermal
IM	Intramuscular
NAbs	Neutralizing antibodies (or neutralizing ADAs)
NAPs	Non-toxic accessory proteins
NTNH	Non-toxic non-hemagglutinin
SC	Subcutaneous
RT	Room temperature
RFU	Relative fluorescence unit
RM ANOVA	Repeated measures analysis of variance
